# Artificial Intelligence in Geospatial Analysis for Flood Vulnerability Assessment: A Case of Dire Dawa Watershed, Awash Basin, Ethiopia

**DOI:** 10.1155/2021/6128609

**Published:** 2021-11-22

**Authors:** Habtamu Tamiru, Megersa O. Dinka

**Affiliations:** ^1^Wollega University, Department of Water Resources and Irrigation Engineering, P.O. Box 395, Nekemte, Ethiopia; ^2^University of Johannesburg, Department of Civil Engineering Sciences, Johannesburg, South Africa

## Abstract

This study presents the novelty artificial intelligence in geospatial analysis for flood vulnerability assessment in Dire Dawa, Ethiopia. Flood-causing factors such as rainfall, slope, LULC, elevation NDVI, TWI, SAVI, K-factor, R-factor, river distance, geomorphology, road distance, SPI, and population density were used to train the ANN model. The weights were generated in the ANN model and prioritized. Initial values were randomly assigned to the NN and trained with the feedforward processes. Ground-truthing points collected from the historical flood events of 2006 were used as targeting data during the training. A rough flood hazard map generated in feedforward was compared with the actual data, and the errors were propagated back into the NN with the backpropagation technique, and this step was repeated until a good agreement was made between the result of the GIS-ANN and the historical flood events. The results were overlapped with ground-truthing points at 88.46% and 89.15% agreement during training and validation periods. Therefore, the application of the GIS-ANN for the assessment of flood vulnerable zones for this city and its catchment was successful. The result of this study can also be further considered along with the city and its catchment for practical flood management.

## 1. Introduction

Flood is one of the natural hazards that happens when either the capacity of the river bank or the infiltration capacity of soil is less than the intensity of the rainfall [1, 2]. This natural hazard occurs when there is torrential rainfall that lasts for a few minutes/hours, resulting in the overflow of the natural river banks. The natural factors and influences of human activities can derive this natural hazard to happen [3]. Floods can harm both properties and human lives [4]. The impacts of the flood are histrionically increasing worldwide [5]. This natural hazard can affect both the residents of urban and rural areas, and the magnitude of the impacts is relatively high in urban areas. According to the global natural disaster reports, over 2.4 billion people have been affected, while about 165,020 people lost their lives due to this natural hazard between the years 2019 and 2020 as approximated by the United Nations (UN). In Africa, over the past two decades of the 2021 century, floods have caused approximately about 280$ billion economic damage. The African countries such as Uganda, Burundi, Djibouti, Kenya, Rwanda, and Somalia have experienced overwhelming floods in the last few years [6–8]. In Ethiopia, which is one of the East African countries, more than 1.1 million people have been affected by flooding. A bunch of studies forecasts and predicts that the severity of this natural disaster would be rise significantly in the climate change scenarios [9–11]. Currently, researchers have been emphasizing a long-term contribution to sustainable disaster risk management using geospatial analysis. The effective flood risk management strategy targets to reduce the loss of human lives and properties in advance of the co-occurrence of the disaster. The reduction and mitigation strategies require high performance and accuracy in spatial analysis. Geospatial analysis for effective flood risk planning is intensively used as a significant tool in handling flood hazards [12, 13]. Due to the absence of geospatial-based flood risk planning in most of the poor countries such as Ethiopia, the impacts of this natural hazard are doubling. Up to recent years, the geographic information system (GIS) and remote sensing (RS) technologies played a vital role in planning for flood disaster risks in urban watersheds [14]. Geospatial technology played a vital role in flood vulnerability assessment for the past few decades and provided the best possible results in making a decision on flood risk management [15]. As the flood risk is a function of spatial analysis, the application of GIS and RS techniques is very significant to flood management strategies. The other capability of GIS is its suitability for the processing of spatially varied physical factors in flood risk assessment. Flood vulnerability analysis and mapping are intensively done by the researchers of natural hazard management in urban catchments as it gives information on the severity of the flood. Geospatial analysis and multicriteria analysis (MCA) are integrated approaches that got international attention in deciding regarding the complicated interrelationship between the physical, social aspects, and economic issues of floods. Pieces of research studies are available in which the MCA and GIS are linked in geospatial analysis. Flood vulnerability analysis is a function of different factors such as hydrologic factors (rainfall, stream power index (SPI), and stream networks), morphometric factors (elevation, slope, landforms, and distance from river), permeability factors (soil type, topographic wetness index (TWI), soil erodibility factor (K), and rainfall erosivity factor (R)), surface dynamics (LULC, soil-adjusted vegetation index (SAVI), normalized difference vegetation index (NDVI)), and anthropogenic influence (population density), and the significance of these factors is prioritized and weights are given in MCA by the method called the analytical hierarchy process (AHP). Nowadays, the application of AHP in prioritizing flood driving factors is lacking its novelty after the application of an artificial begun in areas of geospatial analysis. The artificial neural network (ANN) is a machine-learning approach that uses the processing of the human brain as a basis to develop algorithms that can be used to model complicated relationships among the spatial phenomenon and assess the spatial vulnerability of floods. The ANN is an empirical modelling technique that can solve the complicated relationship between the physical and nonphysical phenomena. Currently, a bunch of research studies is available in which the ANN was used as a modelling tool in areas of hydrological modelling. Wahab and Muhamad Ludin [16] assessed flood vulnerability using the ANN model, and a good result was obtained. It is the novelty of the ANN model that it can easily capture the complicated characteristics of both factual and value-based information that cannot be handled by traditional geospatial techniques. In literature, it was observed that the scholars of hydrological modelling intensively used the ANN model for flood forecasting worldwide [17–19]; however, the application of this newly emerged approach is very rare in areas of geospatial analysis. The current study area (Dire Dawa) is located in the east-central part of Ethiopia, and it is one of the most flood-prone cities when compared to other cities of the country. In Ethiopia, the intensity of rain is very high between June and September for the consecutive three months, and the majority of the flood events were observed in one of these months. In Ethiopian history, the flood event that occurred on 5^th^ and 6^th^ August in 2006 displaced 3000 people and damaged the lives of 200 people in the city (Dire Dawa) over one night. As witnessed by [20, 21], the city was flooded due to the torrential rainfall from upstream highlands. The assessment of flood vulnerability using different criteria such as hydrologic, morphometric, permeability, anthropogenic, and surface dynamic change of the city and its watershed is vital as it can provide information about the spatial severity of the flood. Therefore, this study aimed to present the novelty of the ANN model and geospatial analysis to assess the flood vulnerability in Dire Dawa city, Ethiopia.

## 2. Methods and Materials

### 2.1. Study Area

**Figure 1:**
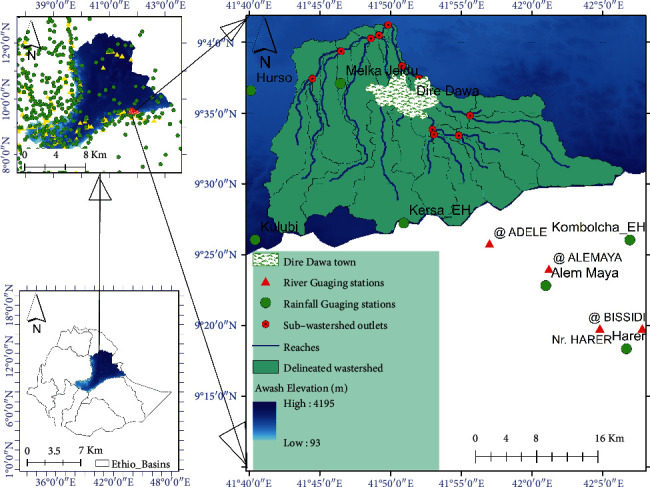
Map of the study area.

The current was conducted in Dire Dawa city, which is one of the cities in Ethiopia. Dire Dawa is situated in eastern-central of Ethiopia, and it is the city which links Ethiopia with Djibouti. The city is geographically located between 9°25′N and 9°45′N latitude and 41°40′E and 42°50′E longitude (Figure 1). The city is surrounded by mountainous areas. The elevation of the entire parts of the city ranges from 1000 to 1600 m above the sea level, and the flood comes from the mountains of the upper catchment. Dhangago is the highest mountain found at the upstream of the city. The urban land of the city is divided by several streams such as Dachatu, Goro, Malka Labu, Laga Hare, and Butuji. These small rivers drain the entire parts of the city. The city is bordered in the north, east, and west by Somali Regional State and in the south and southwest by Oromia Regional State [21]. In this city, a total population of 400,000 was estimated in 2007 and became 466,000 after one year (2008). This city is known as the most flood-prone area when compared to other cities of the country. In history, the 5^th^ and 6^th^ August 2006 were known as black days of the city that very heavy rain caused flooding and both human lives and properties were damaged. According to the national flood report, over 3000 people were displaced, while about 200 people lost their lives.

## 3. Data and Sources

To achieve the objective of this study, data under five criteria, namely, hydrologic criteria, morphometric criteria, permeability criteria, surface dynamic change, and anthropogenic influences were confirmed as flood vulnerability assessing principles, and the detail of these data is presented in Table 1. The data used in this study were derived from the digital elevation model (DEM), river networks, soil map, Landsat images, geology, landforms, rainfall, and population number. The types of data, sources, purpose, spatial, and temporal resolution of the individual each data are provided in Table 1.

## 4. Methodology

**Figure 2:**
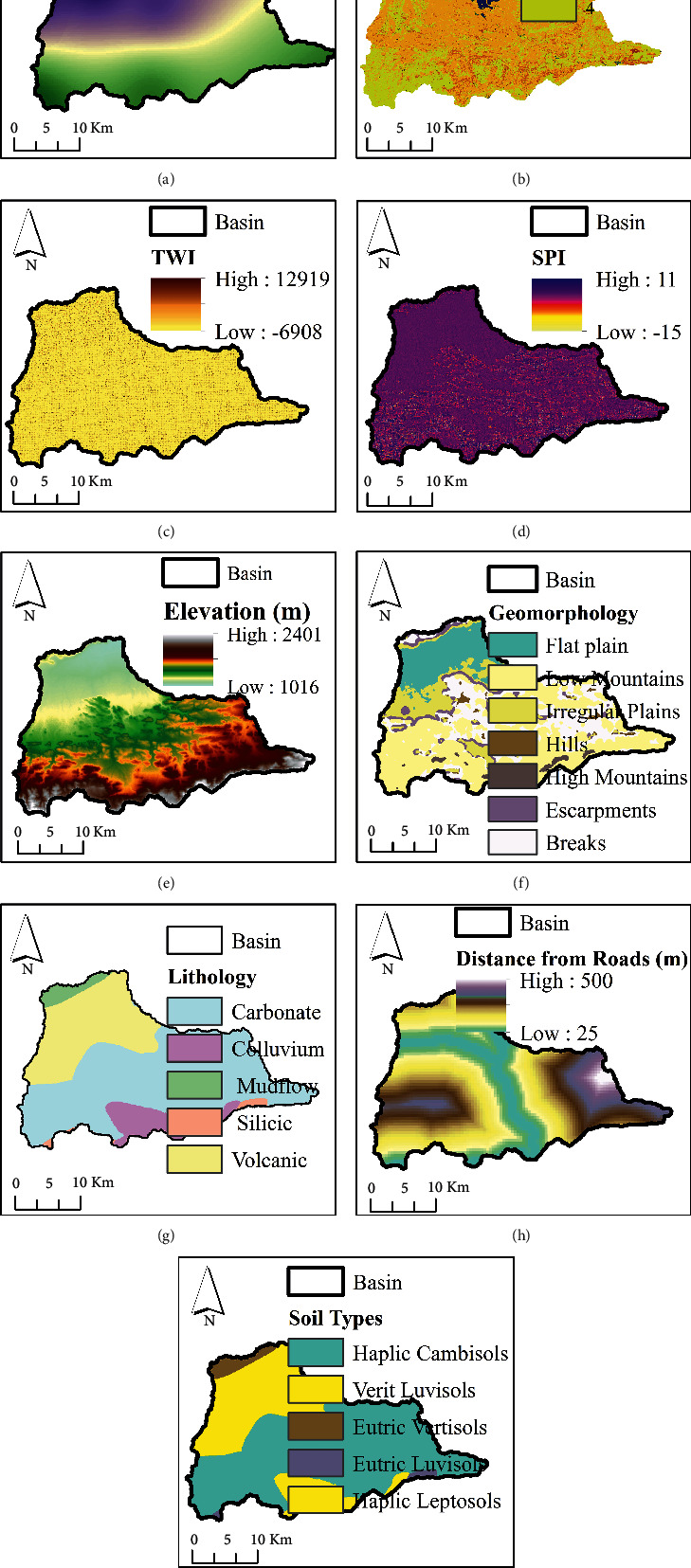
Flood causing factors: (a) rainfall, (b) LULC, (c) TWI, (d) SPI, (e) elevation, (f) geomorphology, (g) lithology, (h) distance from roads, and (i) soil classifications.

**Figure 3:**
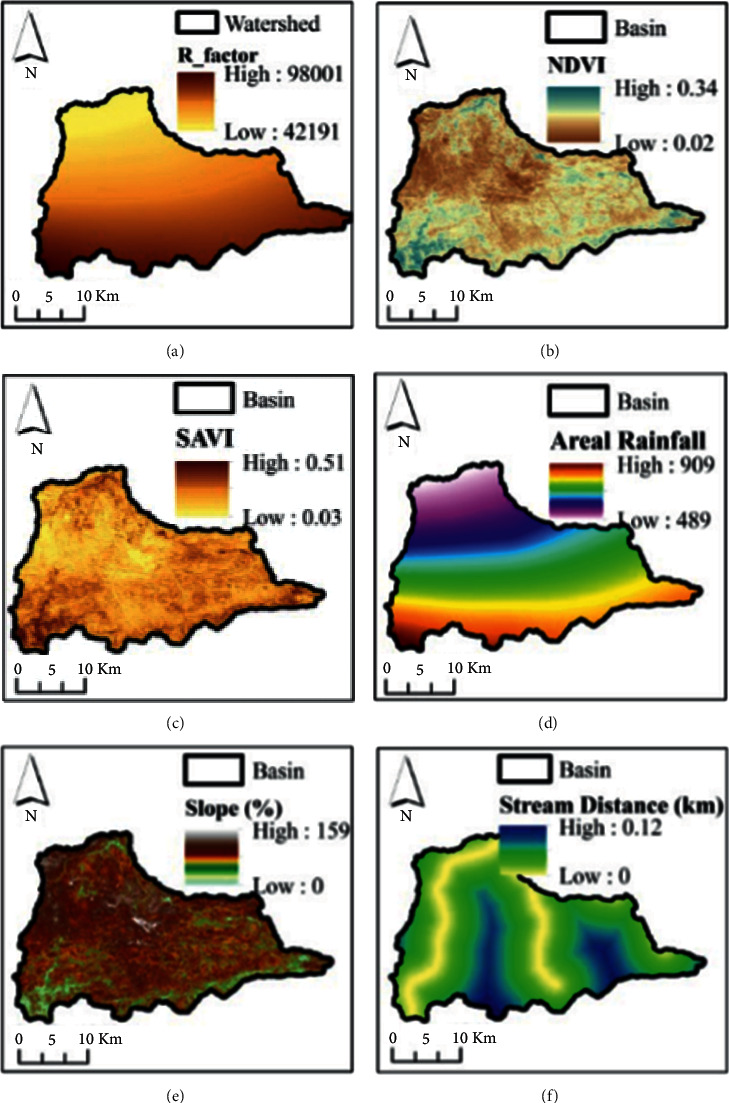
Flood causing factors: (a) R-factor, (b) NDVI, (c) SAVI, (d) areal rainfall, (e) slope, and (f) stream distance from main roads.

This study employed the novelty of an integrated ANN model and geospatial analysis approach [16, 22–24] to assess the vulnerability of flood in Dire Dawa city and its catchments. The assessment of flood vulnerability is a dynamic and complicated phenomenon. The artificial neural network (ANN) is a machine-learning method used to solve complicated and multiple criteria by prioritizing the significance of the individual criteria. The overall procedures used in this study were as follows: selection of flood deriving factors (criteria), preprocessing of the selected criteria and normalizing, selecting the best ANN architecture and setting up the model, assigning random weight values to the selected ANN network, feedforward training, backpropagation, testing the model, and finally generating the flood vulnerability zones using the updated weights in an overlay analysis. The current study uses the ANN multilayer perceptron (ANN-MLP) architecture that consists of three layers; the input layer (the connection between input nodes and hidden nodes), hidden layer (the connection between hidden nodes and output nodes), and output layer (the last node in the network). Before assigning the input data into the input nodes, the derived input for each criterion (the third column in Table 1) was prepared and resampled using a spatial analyst tool in a GIS environment on the same spatial resolution of 30 m × 30 m. The resampled input parameters were normalized, and the values in each pixel were squashed between 0 and 1. The reason why normalization is important is to minimize the training time. Once the values in each pixel of the resampled criterion were normalized using equation (1), the random weights were assigned to networks to start with the training processes.

(1) $$\begin{array}{c} \text{Normalization}=\frac{X-X_{\min}}{X_{\max}-X_{\min}}, \end{array}$$

(2) $$\begin{array}{c} \text{Activation function}=\frac{1}{1+\;e^{x}}, \end{array}$$

where *X* is the normalized input parameters entered into the networks, and *X*_min_ and *X*_max_ are the minimum and maximum values of the normalized input parameters. The raw datasets and the derived input parameters were linked into the NN. The input parameters were selected on the basis of hydrologic, morphometric, permeability, and surface dynamic change and census criteria. Thematic maps were prepared for the individual criteria and prioritized based on their importance of flood vulnerability assessment. The key significant factors of flood vulnerability analysis of the existing data regarding rainfall intensity, land use/land cover (LULC), topographic wetness index (TWI), stream power index (SPI), soil classification, rainfall erosivity factor (R-factor), normalized difference vegetation index (NDVI), soil-adjusted vegetation index (SAVI), population density, areal rainfall, slope, and stream distance of the study area are shown in Figures 2 and 3.

The prioritizing of the selected input parameters was done in the ANN as shown in Figure 3 through weighting the criteria. The final weights of the individual criteria were obtained after training the networks by the method called the backpropagation algorithm. For the training purposes, 26 points were selected randomly (nonflood-prone areas and flood-prone areas), while 20 points of flood events (flood-prone areas) were selected for testing the vulnerability map generated in the ANN. An overlay analysis in the GIS environment was used to redo the generation of the flood vulnerability map until the agreement between the selected points and the flood vulnerability map was made [19, 25, 26].

### 4.1. Flood Vulnerability Assessment in the ANN

**Figure 4:**
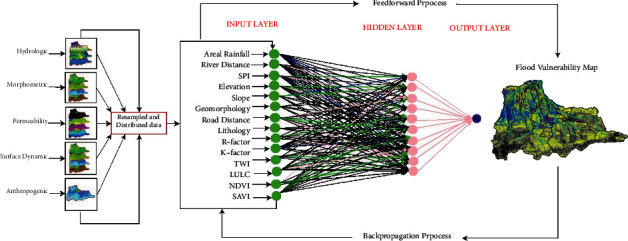
ANN in geospatial analysis for flood vulnerability assessment.

The application of geospatial for flood vulnerability assessment is intensively used worldwide; however, the application of machine-learning (ANN) is still emerging. Up to now, very limited studies are available in which the artificial neural network (ANN) was applied as a flood assessing tool. The hydrologic, morphometric, permeability, surface dynamic change, and the status of the population were used as information to train the ANN model. The random weight values initially assigned to the NN were systematically trained and updated. The information retrieved from the geospatial characteristics of the criteria was reused in the training processes until the derived flood vulnerability map assessed in the GIS-ANN method agreed with the flood events linked into the results based on the ground-truthing points. The general flowchart of fixing the weights for the selected flood vulnerability assessment criteria is shown in Figure 4. As we can see from figure, the geospatial input parameters were linked to the NN, and the weighted sum of the initial weights and input parameters were sent to the hidden nodes for a further process called activation (equation (2)). After hidden nodes, the networks again send the weighted sum of activated values to the output nodes, and at this stage, a rough result is obtained. Once the rough result is obtained in the final node (output layer), the backpropagation process will continue to reduce the difference between the rough result obtained in NN and the target values. At this stage, the pixel-to-pixel values of the roughly generated flood vulnerability map in the ANN are compared to the map generated based on flood events (ground-truthing values) recorded in the city. The NN keeps training until an agreement is made between the ANN result and ground-truthing values. The feedforward process receives the updated values of weights from the backpropagation, and this will continue till both results (GIS-ANN and ground-truthing) were agreed upon. After training, the result was further evaluated and checked by other floodplain areas.

**Figure 5:**
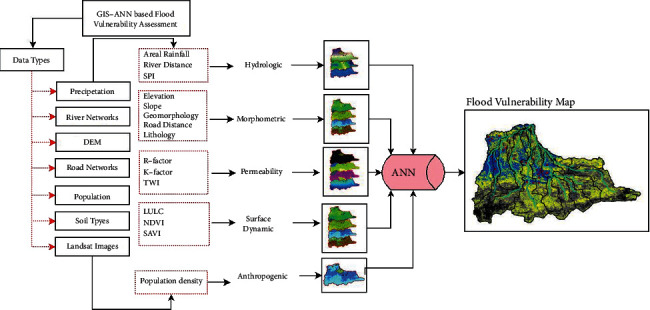
General flowchart of the study.

To check the performance of the GIS-ANN method in assessing flood vulnerability, the result obtained in the GIS-ANN and the floodplain areas identified for the testing purpose were compared based on the pie chart plots and overlapping the result with ground-truthing points. The pie chart plots are based on plotting the degree of importance of each criterion with the flood levels generated in the GIS-ANN, where the visualization is based on the percentage of overlapping (equation (3)) points between the generated flood vulnerable areas and flood events (points). The detailed training process implemented in this study is shown in Figure 5.

Points were overlapped with the flood vulnerable areas, and the overlapped points were counted. The ratio of the number of the counted points to the total number of the points was calculated by the intersection analyst tool in the GIS environment [27].

(3) $$\begin{array}{c} \text{Overlapping percentage }\left(\%\right)=\frac{\text{Overlapped number of points}}{\text{Total number of points}}. \end{array}$$

## 5. Results and Discussion

**Figure 6:**
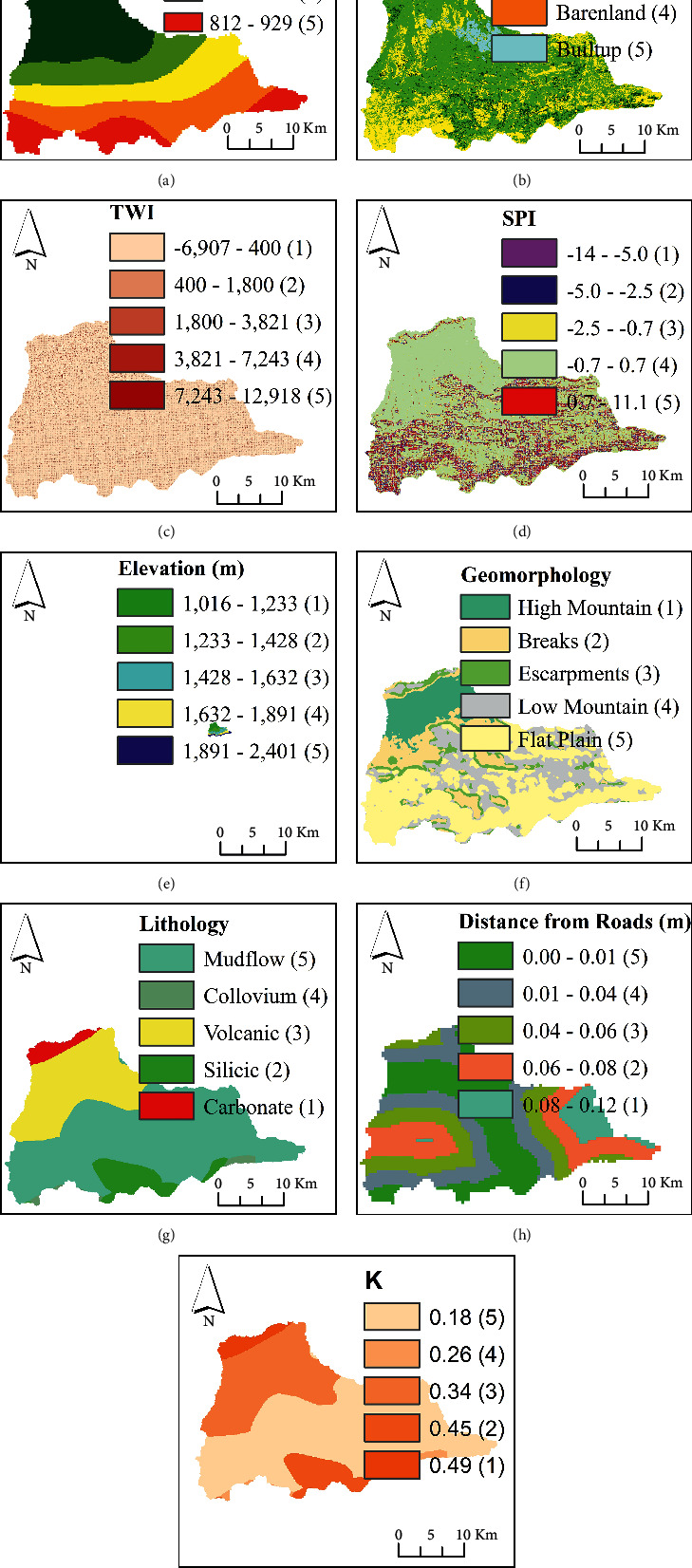
Reclassified flood causing factors based on flood vulnerability assessment: (a) rainfall, (b) LULC, (c) TWI, (d) SPI, (e) elevation, (f) geomorphology, (g) lithology, (h) distance from roads, and (i) K-factor.

**Figure 7:**
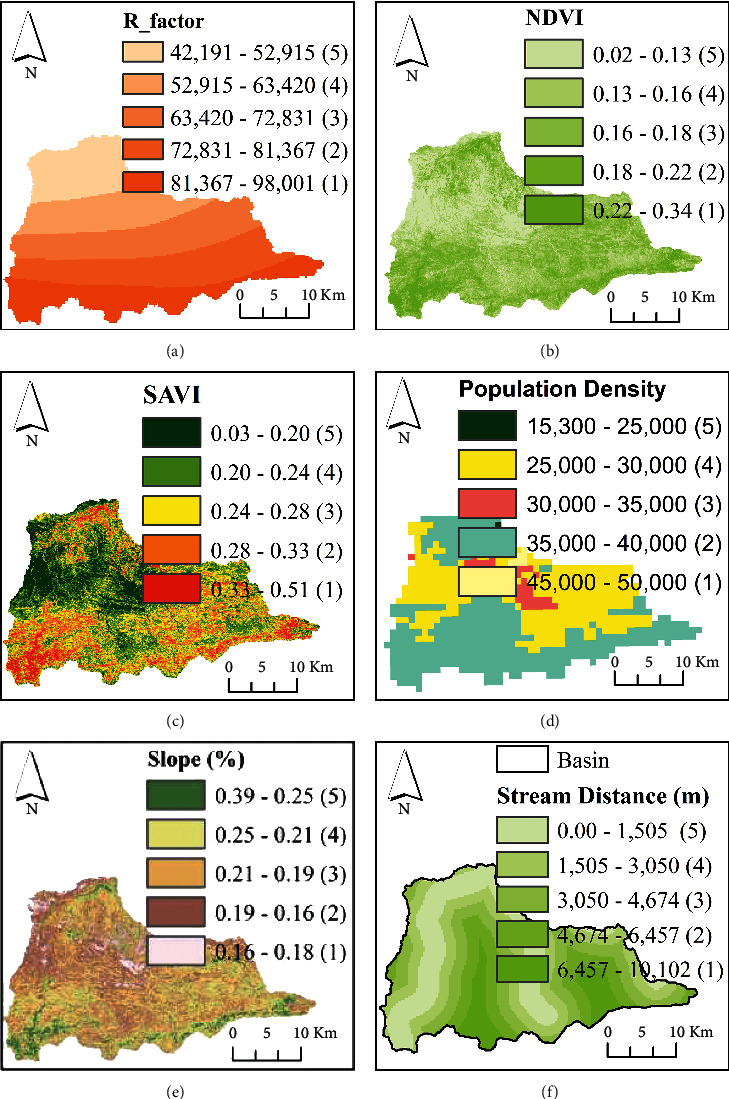
Reclassified flood causing factors based on flood vulnerability assessment: (a) R-factor, (b) NDVI, (c) SAVI, (d) population density, (e) slope, and (f) stream distance.

In this study, the novelty of artificial intelligence in geospatial analysis for flood vulnerability assessment in Dire Dawa watershed, Awash basin, Ethiopia, is presented. The current study used flood causing factors such as hydrologic factors (rainfall, river distance from the settlement, and stream power index), morphometric factors (elevation, slope, geomorphology, and distance of roads from rivers), permeability factors (soil erodibility factor, rainfall erosivity, and topographic wetness index), surface dynamic factors (land use/land cover, normalized difference vegetation index, and soil-adjusted vegetation index), and census status (population density around the flood-prone areas) for the assessment of flood vulnerable areas in Dire Dawa city and its catchments. The raw datasets were processed in a GIS environment and sent to the ANN model to prioritize (weighting) based on their importance for the assessment of flood vulnerable areas. The weights of the individual criteria selected in the study were fixed based on the feedforward and backpropagation processes. The reclassified maps of flood-causing factors derived from the existing data (Figures 2 and 3) are shown in Figures 6 and 7.

**Figure 8:**
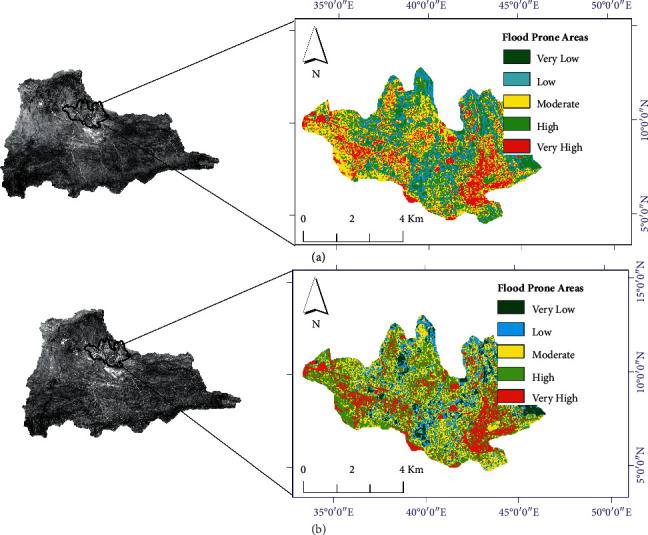
Identified flood prone areas in Dire city: (a) flood events and (b) ANN results.

**Figure 9:**
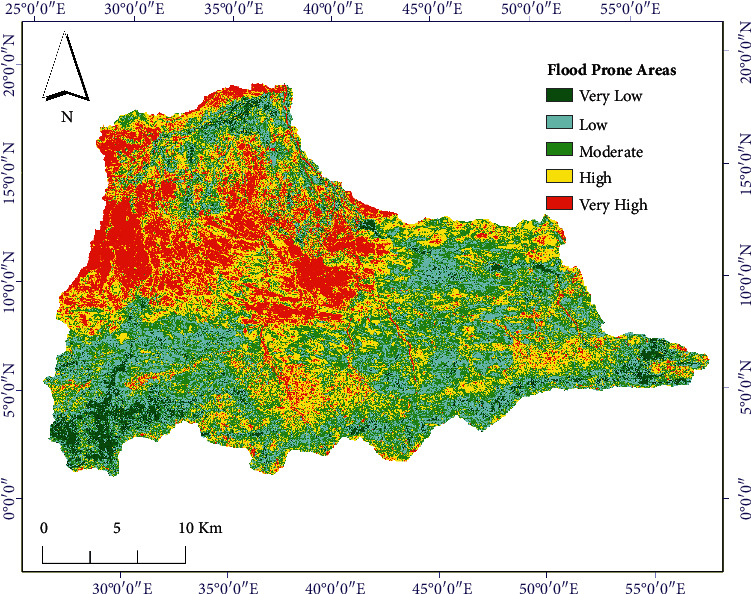
Identified flood prone areas in Dire Dawa watershed.

As we can see from the reclassified maps, the categories or classifications presented in all maps is based on the studies conducted in different parts of the world and consulted in different pieces of literature [4, 9, 28, 29]. Random values of weights were assigned to the NN. The current study used the ANN multilayer perceptron (ANN-MLP) architecture as shown in Figure 4. The training process was started with the initial random values of weights assigned in R programming and activated in the hidden nodes with the sigmoid activation function (equation (2)). With the initial values of weights, a rough flood vulnerable map was obtained from the first feedforward training as shown in Figure 5 and compared to the ground-truthing points, and the error was propagated back into the NN. During the training processes, two major activities were simultaneously performed: the NN improves the weight assigned for the individual criteria and flood vulnerable (flood-prone areas) was generated using the improved weights in the GIS environment with the overlay analyst tool. In the same fashion, the second round of the training process was performed, and flood-prone areas were generated. These steps were repeated until the final map, as shown in Figure 5, which is the best map when compared to the historical flood points. The weights generated in the ANN model were used to prioritize the individual factor. Wahab and Muhamad Ludin [16] ranked the key significant factors and assigned percentage for each flood-prone classification. For this specific study, rainfall, slope, elevation, and LULC revealed the most significant for the assessment of flood hazard zones, and factors such as rainfall erosivity factor (R-factor), soil erodibility factor (K-factor), topographic wetness index (TWI), stream power index (SPI), normalized difference vegetation index (NDVI), soil-adjusted vegetation index (SAVI), and road distance from the settlement showed high to moderate significance, whereas the remaining flood deriving factors such as geomorphology and census status relatively showed less significance. The vulnerable areas were identified using the weights updated in the ANN. The final updated values of weights were used in overlay analysis in the GIS environment. Sarkar and Mondal [4] used an overlay analysis to generate flood vulnerable areas with the values prioritized in the AHP technique, and five flood hazard zones were identified (Figure 8). The result obtained in this study was evaluated using ground-truthing points collected in the floodplain. Historical flood events and the information obtained from the local respondents as detailed in [21] were used to check the GIS-ANN model results. From the 2006 flood events, about 26 ground points were marked and linked with the model results. The flood indicating zones generated in the GIS-ANN approach was overlapped with the actual point data. The historical flood events of 2006 were added to the GSI-ANN result map. First, the flood hazard zones were generated at the catchment level and extracted for the city. The general flood vulnerable zones identified at the catchment and city level are shown in Figure 9.

**Figure 10:**
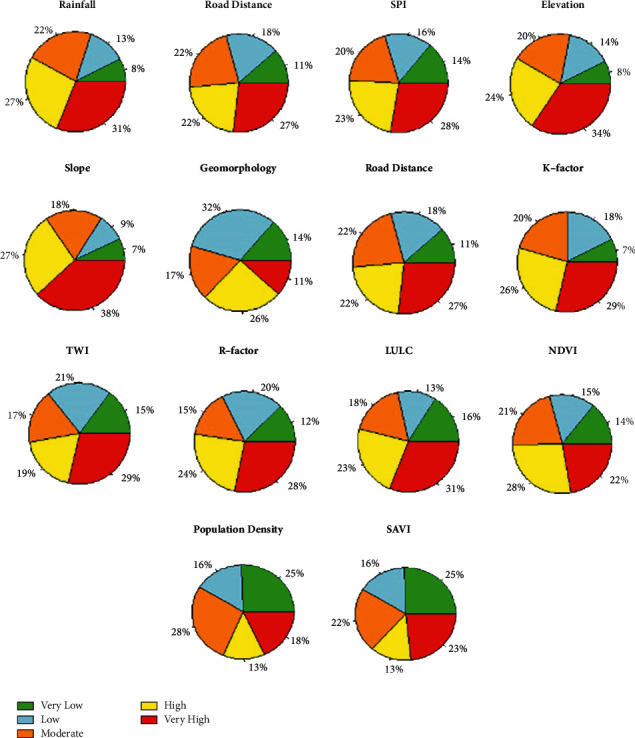
The percentage of importance of flood causing factors.

Five qualitative-based flood vulnerable zones were identified in this study, as shown in Figure 9. As we can see from this figure, flood-prone areas based on the severity of the flood as very high (red), high (yellow), moderate (light-green), low (light-blue), and very low (green) were identified. The corresponding percentage of the vulnerable areas to the qualitative classifications are 18.21%, 32.73%, 35.94%, 7.25%, and 5.87%, respectively. The significance of each individual factors of flood causing parameters is shown in Figure 10.

The areas under floodplain are due to the torrential rainfall from the upstream of the city. As we have already described the topographic characteristics of the current study area, the upper part of the catchment is surrounded by mountainous regions. As a witness to the flood events of 2006 [21, 30], the city was flooded as a result of the heavy rain from the mountainous areas. The results obtained in the GIS-ANN method were overlapped with the ground-truthing points for further evaluation. A total of 26 points were collected from floodplains; 88.46% (23 points) and 89.15% were fully overlapped with the generated flood hazard zones under very high flood levels during the training and validation periods, respectively.

## 6. Conclusion

In this study, the novelty of artificial intelligence in geospatial analysis for flood vulnerability assessment in Dire Dawa watershed, Awash basin, Ethiopia, is presented. To achieve the objective of the study, five major criteria such as hydrologic criteria (rainfall, stream power index (SPPI), and river Euclidean distance from settlement), morphometric criteria (elevation, road distance from the floodplains, slope, and geomorphology), soil permeability criteria (soil erodibility factor (K-factor), topographic wetness index (TWI), and rainfall erosivity factor (R-factor)), surface dynamic charge criteria (LULC, normalized difference vegetation index (NDVI), and soil-adjusted vegetation index (SAVI)), and census status (population density) were confirmed as flood causing factors to train the ANN model. The raw datasets of the selected flood causing factors were preprocessed and resampled in a spatial analyst tool in a GIS environment and distributed over 30 m × 30 m spatial resolution. The significance of the selected flood causing factors was prioritized in the ANN model with feedforward and backpropagation training processes. The neural networks (NNs) were initially assigned by random values of weights and started the feedforward training processes. With the initial values of weights and feedforward training processes, rough flood hazard zones were prepared, and at this stage, the error was very high. The backpropagation process was started targeting the ground-truthing points collected from the historical flood events, and the process was repeated until the error between the GIS-ANN result and the actual data mage an agreement. Accordingly, flood vulnerable zones classified under five flood levels, namely, very low, low, moderate, high, and very high were identified. The performance of GIS-ANN results was further evaluated by overlapping the ground-truthing points, and about 88.46% agreement was made. Among the flood causing criteria selected for the assessment of flood vulnerable zones, LULC, rainfall, elevation, and slope are the most important, and this is due to the topographic condition of the city. Therefore, the application of an integrated artificial intelligence and geospatial analysis for the assessment of flood vulnerable zones was successful.

## Figures and Tables

**Table 1:** Detailed data and sources used in the study.

S/N	Raw data	Derived criteria	Sources	Spatial/Temporal resolution	Purposes
1	Precipitation (mm)	Rainfall	National Meteorology Agency	Dire Dawa and its watershed for 2015–2020 periods	To derive rainfall erosivity factor (*R*) and areal rainfall distribution (kriging)

2	Landsat 8 OLI/TIRS/C2L2	LULC		Dire Dawa and its watershed for 2006, 2008 and 2021	To generate land use/land cover for the watershed (supervised classification)

3	Landsat 8 OLI/TIRS/C2L2 (B4 and B5)	SAVI	https://earthexplorer.usgs.gov/	Dire Dawa and its watershed	To generate information about soil influences on causing flooding

4	Landsat 8 OLI/TIRS/C2L2 (B3 and B5)	NDVI		https://earthexplorer.usgs.gov/	To quantify the vegetation along the flood-prone areas

5	Soil types	K	Ministry of Water, Irrigation, and Energy (MoWIE)		To derive soil erodibility factor (K)

6		TWI			To identify hydrological flow path

7		SPI			To measure the erosive power of the flooding

8	DEM (12.5 × 12.5 m)	E	https://search.asf.Alaska.edu/	Dire Dawa and its watershed	To generate the differences between the consecutive points in the watershed

9		S			To derive the surface differences

10	River networks	SD	Ministry of Water, Irrigation, and Energy (MoWIE)		To derive the Euclidean distance between the settlements and main river course

11	Landforms	G	geoportal.rcmrd.org/layers/servir%3Aafrica_landforms		To generate the physical/geographical of the city and its watershed

13	Road networks	RD	Digitized from Google Earth and processed in GIS environment		To derive the Euclidean distance between the settlements and flood-prone areas

14	Census data	PD	Central Statistical Agency (CSA)	2006, 2008, 2021	To identify the settlement with dense population in the city and its watershed

## Data Availability

All data generated during the manuscript analysis are included within the article. Furthermore, datasets are available from the corresponding author upon request.

## References

[1] Andualem T. G., Hagos Y. G., Kefale A., Zelalem B. (2020). Soil erosion-prone area identification using multi-criteria decision analysis in Ethiopian highlands. *Modeling Earth Systems and Environment*.

[2] Dembélé M., Zwart S. J. (2016). Evaluation and comparison of satellite-based rainfall products in Burkina Faso, West Africa. *International Journal of Remote Sensing*.

[3] Vijay R., Samal D., Mohapatra P. K. (2011). GIS based identification and assessment of groundwater quality potential zones in Puri city, India. *Journal of Water Resource and Protection*.

[4] Sarkar D., Mondal P. (2020). Flood vulnerability mapping using frequency ratio (FR) model: a case study on Kulik river basin, Indo-Bangladesh Barind region. *Applied Water Science*.

[5] Moreno J. M., Sánchez J. M., Espitia H. E. (2020). Use of computational intelligence techniques to predict flooding in places adjacent to the Magdalena River. *Heliyon*.

[6] Lohani A. K., Kumar R., Singh R. D. (2005). Flood forecasting using artificial neural networks Hydrological time series modeling: a comparison between adaptive neuro-fuzzy, neural network and autoregressive techniques. *Journal of Hydrology*.

[7] Risi R. D., Jalayer F., Paola F. D., Carozza S. (2020). From flood risk mapping toward reducing vulnerability: the case of Addis Ababa. *Natural Hazards*.

[8] Shrestha B. B., Kawasaki A. (2020). International Journal of Disaster Risk Reduction Quantitative assessment of flood risk with evaluation of the effectiveness of dam operation for flood control: a case of the Bago River Basin of Myanmar. *International Journal of Disaster Risk Reduction*.

[9] Desalegn H., Mulu A. (2021a). Flood vulnerability assessment using GIS at Fetam watershed, upper Abbay basin, Ethiopia. *Heliyon*.

[10] Mandal B., Dolui G., Satpathy S. (2018). Land suitability assessment for potential surface irrigation of river catchment for irrigation development in Kansai watershed, Purulia, West Bengal, India. *Sustainable Water Resources Management*.

[11] Mohamed C., Shantha N. (2019). International Soil and Water Conservation Research Soil loss estimation using rusle model to prioritize erosion control in KELANI river basin in Sri Lanka. *International Soil and Water Conservation Research*.

[12] Ali S. A., Ahmad A. (2020). Suitability analysis for municipal landfill site selection using fuzzy analytic hierarchy process and geospatial technique. *Environmental Earth Sciences*.

[13] Das S. (2019). Geospatial mapping of flood susceptibility and hydro-geomorphic response to the floods in Ulhas basin, India. *Remote Sensing Applications: Society and Environment*.

[14] Gedam K., Dagalo S. (2020). Journal of hydrology: regional studies identification of groundwater potential zones using Proxy data: case study of megech watershed, Ethiopia. *Journal of Hydrology: Regional Studies*.

[15] Rajaveni S. P., Brindha K., Elango L. (2017). Geological and geomorphological controls on groundwater occurrence in a hard rock region. *Applied Water Science*.

[16] Wahab A. M., Muhamad Ludin A. N. (2018). Flood vulnerability assessment using artificial neural networks in Muar Region, Johor Malaysia. *IOP Conference Series: Earth and Environmental Science*.

[17] Chan V. K. H., Chan C. W. (2020). Towards explicit representation of an artificial neural network model: comparison of two artificial neural network rule extraction approaches. *Petroleum*.

[18] Gholami V., Khaleghi M. R. (2021). A simulation of the rainfall-runoff process using artificial neural network and HEC-HMS model in forest lands. *Journal of Forest Science*.

[19] Rezaeianzadeh M., Tabari H., Arabi Yazdi A., Isik S., Kalin L. (2014). Flood flow forecasting using ANN, ANFIS and regression models. *Neural Computing & Applications*.

[20] Erena S. H., Worku H. (2018). Flood risk analysis: causes and landscape based mitigation strategies in Dire Dawa city, Ethiopia. *Geoenvironmental Disasters*.

[21] Erena S. H., Worku H. (2019). Urban flood vulnerability assessments: the case of Dire Dawa city, Ethiopia. *Natural Hazards*.

[22] Mallick J. (2021). Municipal solid waste landfill site selection based on fuzzy‐ahp and geoinformation techniques in ASIR region Saudi Arabia. *Sustainability*.

[23] Ogato G. S., Bantider A., Abebe K., Geneletti D. (2020). Geographic information system (GIS)-Based multicriteria analysis of flooding hazard and risk in Ambo Town and its watershed, West shoa zone, oromia regional State, Ethiopia. *Journal of Hydrology: Regional Studies*.

[24] Tolche A. D. (2020). Groundwater potential mapping using geospatial techniques: a case study of Dhungeta-Ramis sub-basin, Ethiopia. *Geology, Ecology, and Landscapes*.

[25] Veintimilla-Reyes J., Cisneros F., Vanegas P. (2016). Artificial neural networks applied to flow prediction: a use case for the tomebamba river. *Procedia Engineering*.

[26] Young C. C., Liu W. C., Wu M. C. (2017). A physically based and machine learning hybrid approach for accurate rainfall-runoff modeling during extreme typhoon events. *Applied Soft Computing Journal*.

[27] Wang Y., Liu Z., Liao H. (2017). Improving the performance of gis polygon overlay computation with mapreduce for spatial big data processing improving the performance of gis polygon overlay computation with mapreduce for spatial big data processing. *Cluster Computing*.

[28] Feloni E., Mousadis I., Baltas E. (2020). Flood vulnerability assessment using a GIS-based multi-criteria approach—the case of Attica region. *Journal of Flood Risk Management*.

[29] Leal M., Reis E., Pereira S., Santos P. P. (2021). Physical vulnerability assessment to flash floods using an indicator-based methodology based on building properties and flow parameters. *Journal of Flood Risk Management*.

[30] Erena S. H., Worku H., De Paola F. (2018). Flood hazard mapping using FLO-2D and local management strategies of Dire Dawa city, Ethiopia. *Journal of Hydrology: Regional Studies*.

